# Integrative Role of Hyperbaric Oxygen Therapy on Healthspan, Age-Related Vascular Cognitive Impairment, and Dementia

**DOI:** 10.3389/fragi.2021.678543

**Published:** 2021-09-23

**Authors:** Priya Balasubramanian, Jordan Delfavero, Adam Nyul-Toth, Amber Tarantini, Rafal Gulej, Stefano Tarantini

**Affiliations:** ^1^ Vascular Cognitive Impairment and Neurodegeneration Program, Department of Biochemistry and Molecular Biology, University of Oklahoma Health Sciences Center, Oklahoma City, OK, United States; ^2^ International Training Program in Geroscience, Institute of Biophysics, Biological Research Centre, Eötvös Loránd Research Network (ELKH), Szeged, Hungary; ^3^ International Training Program in Geroscience, Doctoral School of Basic and Translational Medicine/Department of Public Health, Semmelweis University, Budapest, Hungary; ^4^ Department of Health Promotion Sciences, College of Public Health, University of Oklahoma Health Sciences Center, Oklahoma City, OK, United States

**Keywords:** aging, neurovascular coupling, neurodegeneration, geroscience, aging, dementia, cognitive function

## Abstract

Growing life expectancy will contribute to the on-going shift towards a world population increasingly comprised of elderly individuals. This demographic shift is associated with a rising prevalence of age-related diseases, among all age-related pathologies it has become crucial to understand the age-associated cognitive changes that remain a major risk factor for the development of vascular cognitive impairment and dementia (VCID). Furthermore, age-related Alzheimer’s disease and other neurogenerative diseases with vascular etiology are the most prominent contributing factors for the loss of cognitive function observed in aging. Hyperbaric Oxygen Therapy (HBOT) achieves physiologic effects by increasing oxygen tension (PO2), raising oxygen tissue levels, decreasing intracranial pressure and relieving cerebral edema. Many of the beneficial effects of HBOT exert their protective effects at the level of the microcirculation. Furthermore, the microcirculation’s exquisite pervasive presence across every tissue in the body, renders it uniquely able to influence the local environment of most tissues and organs, including the brain. As such, treatments aimed at restoring aging-induced functional and structural alterations of the cerebral microcirculation may potentially contribute to the amelioration of a range of age-related pathologies including vascular cognitive impairment, Alzheimer’s disease, and vascular dementias. Despite the presented evidence, the efficacy and safety of HBOT for the treatment of age-related vascular cognitive impairment and dementia remains understudied. The present review aims to examine the existing evidence indicative of a potential therapeutic role for HBOT-induced hyperoxia against age-related cerebromicrovascular pathologies contributing to cognitive impairment, dementia and decreased healthspan in the elderly.

## Introduction

The number of individuals over the age of 65 is expected to increase by more than 50 percent before 2050. In the United States alone, the existing population of 40 million elderlies will grow to nearly 90 million within the next 30 years (Harada et al., 2013). Growing life expectancy will contribute to the on-going shift towards a world population increasingly comprised of elderly individuals. This demographic shift is associated with a rising prevalence of age-related diseases (Jaul and Barron, 2017; Yabluchanskiy et al., 2018). Among all age-related pathologies it has become crucial to understand the age-associated cognitive changes that remain a major risk factor for the development of vascular cognitive impairment and dementia (VCID). Furthermore, age-related Alzheimer’s disease and other neurodegenerative diseases with vascular etiology are the most prominent contributing factors for the loss of cognitive function observed in aging (Schneider et al., 2007; Felsky et al., 2019) and at the same time play a crucial role in the quality of life of older adults and impose a significant financial burden on our society (Dallmeyer et al., 2017).

The brain receives between 15 and 20% of the cardiac output under resting conditions. To carry out its critical functions, and maintain cognitive abilities, the brain requires a constant supply of oxygen and nutrients as well as adequate cerebral blood flow (CBF) for the washout of metabolic by-products generated by actively firing neurons and glia populations. Cerebral metabolism is highly active and requires 20% of the body’s available oxygen while the brain represents only 2% of an individual’s total mass. Without enough oxygen or with low blood-oxygen levels, the onset of hypoxia and hypoglycemia pose a significant threat to brain function, resulting in cellular injury and neurodegeneration (Falkowska et al., 2015). In humans as well as in all mammals, the oxygen storage capacity in the central nervous system is limited, and even momentary interruptions in the oxygen supply rapidly impair neuronal function (Angelova et al., 2015). To prevent ischemic damage, stimulus-evoked changes in neural activity are closely coupled to metabolism, cerebral blood oxygenation, and dynamic modulation of CBF under normal physiological conditions. This feed-forward mechanism termed neurovascular coupling (NVC) matches CBF, which supplies the brain with oxygen and nutrients, to the metabolic needs of activated neurons (Iadecola, 2004; Park et al., 2007; Tarantini et al., 2017a; Ungvari et al., 2017a; Tarantini et al., 2017b).

Clinical and experimental studies have revealed an age-related functional impairment of the neurovascular unit, which likely contributes to neurovascular dysfunction and cognitive decline in aging and in age-related neurodegenerative diseases (Tarantini et al., 2017c). Hyperbaric oxygen therapy (HBOT) has been utilized in the treatment of a multitude of medical conditions since its first documentation in 1662 (Aero-chalinos, 1664) and then widely used during the 19th century to treat for decompression sickness (Al-Waili et al., 2005). This non-invasive highly translatable therapy is delivered by a procedure in which 100% pure oxygen is administered at greater than atmospheric pressure. The typical pressures used to administer HBOT range between 2 and 3 atmospheres absolute (ATA) for 60–120 min, which is 2–3 times what is normally experienced at sea level (Kranke et al., 2015). HBOT, as an adjuvant treatment, has been widely researched in models of cerebrovascular injury such as stroke and has shown great promise for improving the recovery time and reducing the disability rate (Zhai et al., 2016). Because both ischemic and hemorrhagic strokes result in regional hypoxia which imposes a major pathological stress in the brain, HBOT achieves physiologic effects by increasing oxygen tension (PO_2_), raising oxygen tissue levels, decreasing intracranial pressure and relieving cerebral edema (Sukoff and Ragatz, 1982). Many of the beneficial effects of HBOT exert their protective effects at the level of the microcirculation. Furthermore, the microcirculation’s exquisite pervasive presence across every tissue in the body renders it uniquely able to influence the local environment of most tissues and organs, including the brain. As such, treatments aimed at restoring aging-induced functional and structural alterations of the cerebral microcirculation may potentially contribute to the amelioration of a range of age-related pathologies including vascular cognitive impairment, Alzheimer’s disease, and vascular dementias.

Despite the presented evidence, the efficacy and safety of HBOT for the treatment of age-related vascular cognitive impairment and dementia remains understudied. The present review aims to examine the existing evidence indicative of a potential therapeutic role for HBOT-induced hyperoxia against age-related cerebromicrovascular pathologies contributing to cognitive impairment, dementia, and decreased healthspan.

## Mechanisms of HBOT in the Vasculature: Implications for Healthspan and Cognitive Function

### Vascular Mechanisms

**FIGURE 1:**
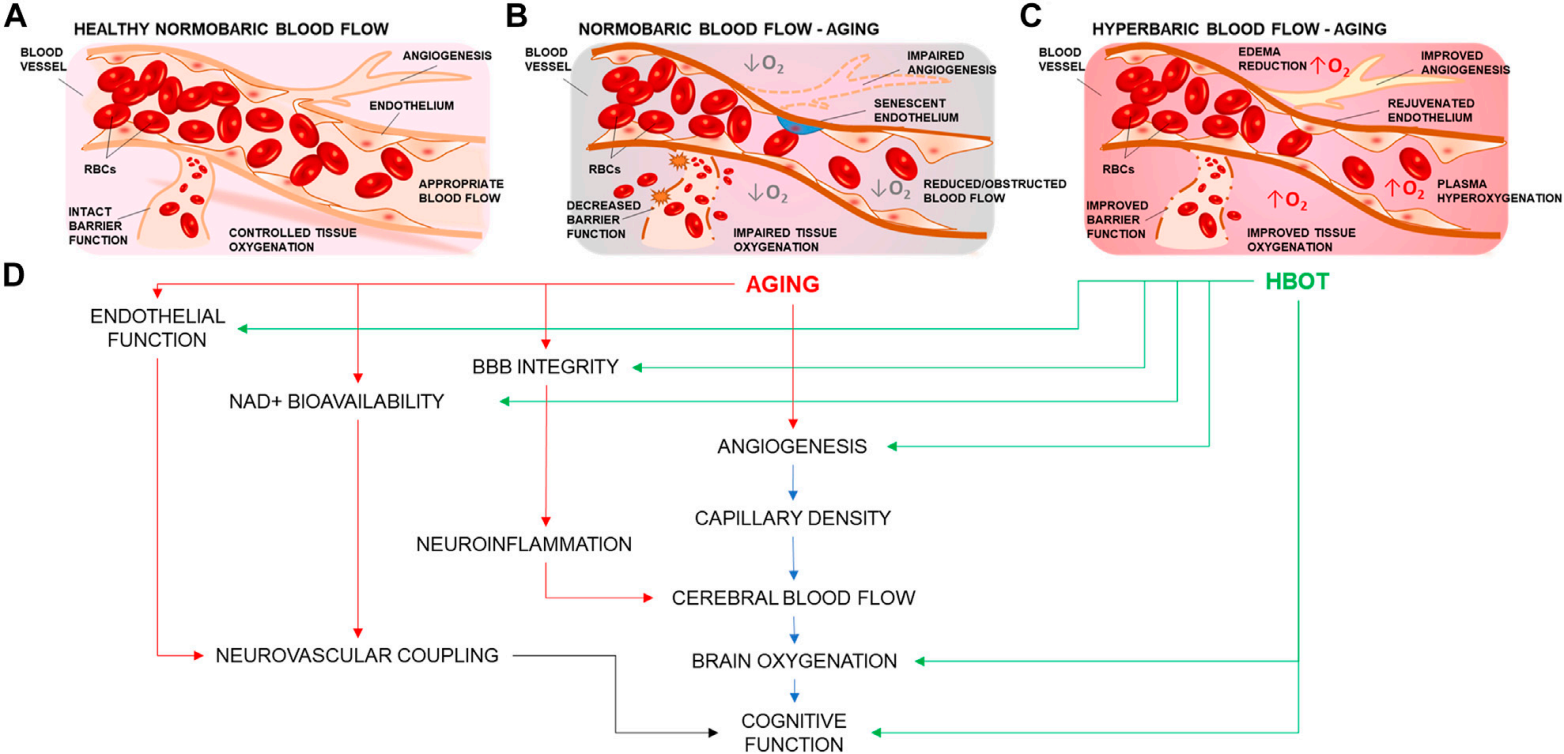
Summary representation of the cerebrovascular effects of hyperbaric oxygen treatment. **(A)** Representation of a branching cerebral arteriole. In young healthy individuals, inhaled 21% O_2_ is sufficient to ensure adequate brain oxygenation. **(B)** Age-related cerebromicrovascular disease is associated with increased BBB permeability, neuroinflammation, declining endothelial function, mitochondrial dysfunction, oxidative stress, loss of Nrf2 activity, increase in senescent cell burden, and NAD+ depletion **(C)** Administration of 100 percent oxygen in a pressurized environment results in hemoglobin saturation and hyperoxygenated plasma Hyperoxygenation exerts multiple beneficial effects that ameliorate and reverse brain microvascular pathologies. **(D)** HBOT targets many of the age-related impairments in vascular mechanisms that drive regulation of blood flow and cognition.

Treatments and therapies that aim to improve vascular and cerebrovascular health have been associated with increased health-span (period of healthy living) in aging (Brown et al., 2007; Drummond et al., 2011; Seals et al., 2016). Administration of 100 percent oxygen to a patient in a pressurized environment results in hemoglobin saturation. Unbound extra oxygen can dissolve in the plasma producing hyperoxygenated blood (Goyal et al., 2019), so that the dissolved fraction becomes the main source of O_2_ available to cells (Demchenko et al., 2005). Hyperoxygenation together with higher atmospheric pressure have been observed to exert multiple effects on the brain and its cerebrovasculature (Figure 1), ranging from restoration of blood brain barrier (BBB) permeability (Li et al., 2018a), improved angiogenesis (Johnson and Wilgus, 2014), edema reduction (Sukoff and Ragatz, 1982; Yagishita et al., 2017), to the modulation of perceived painful stimuli (Sutherland et al., 2016). Cerebromicrovascular mechanisms are also extensively studied in the context of age-related vascular dementias. It is well recognized that age-related microvascular rarefaction (Tucsek et al., 2014), increased BBB permeability (Li et al., 2018a), endothelial dysfunction (Herrera et al., 2010; Ungvari et al., 2018a), inflammation (Csiszar et al., 2014; Ungvari et al., 2018b; Fulop et al., 2018), and impairment of neurovascular coupling responses (Tarantini et al., 2017c) are among critical factors in the development of cerebromicrovascular pathologies associated with neurodegeneration and loss of cognitive function which are responsible for loss of quality of life and diminished healthspan in aging.

Over the past decades a growing number of studies have implicated HBOT for the treatment of vascular dementias through the following possible mechanisms: increased oxygen supply and tissue oxygen partial pressure (pO_2_), relieving cerebral edema (Sukoff and Ragatz, 1982), decreasing intracranial pressure, promoting tissue healing and angiogenesis (Johnson and Wilgus, 2014), improved metabolism, reduced apoptosis, alleviating oxidative stress, increased mitochondrial function and promoting cell differentiation and regeneration (Robertson and Hart, 1999; Feldmeier, 2003; Wang et al., 2016) (Figure 1). During inhalation of normobaric air, arterial pO_2_ is approximately 100 and 55 mmHg in the tissues. However when breathing 100 percent oxygen at 3 ATA arterial pO_2_ can increase to about 2,000 mmHg and to 500 mmHg in the tissues (Tibbles and Edelsberg, 1996). By exceeding the oxygen carrying capacity of hemoglobin, the oxygen in solution saturates the blood plasma and can better oxygenate areas where red blood cells cannot reach or where hemoglobin oxygen carriage is impaired (Figure 1C), such as in microvascular injury or carbon monoxide poisoning (Leach et al., 1998; Phillips, 2000).

HBOT not only provides increased access of oxygen to damaged tissue via plasma saturation, but also encourages new blood vessel formation by activation transcription factors such as VEGF (Goyal et al., 2019). Although the mechanisms are not fully understood, HBOT is a well-established treatment for decompression sickness. This condition affects individuals that experience trauma, surgery, or deep-sea diving, resulting in the formation of air embolisms. Additional therapeutic targets that deserve more investigation include cognition. As such, recent studies have aimed to investigate the cellular and molecular mechanisms underlying HBOT to better understand its potential clinical role in age-related cognitive impairment. HBOT is particularly effective in wound healing, it works by amplifying the oxygen diffusion gradients along ischemic wounds, promoting extracellular matrix synthesis required for angiogenesis (Knighton et al., 1981; Hunt, 1988).

### Microvascular Mechanisms

In the cerebral microvasculature, age-related pathological changes include loss of vascular density (Tucsek et al., 2014) and decreased angiogenesis (Lin et al., 2018), increased incidence of ischemic and hemorrhagic microvascular injury (Ungvari et al., 2017b), and neuroinflammation which is associated with loss of cognitive function. The physiological basis and documented effects of HBOT as it was studied on peripheral vasculature and tissues could be applied to the cerebral microvasculature to study the potential therapeutic effects of hyperoxia against age-related vascular cognitive impairment. In healthy vasculature hyperoxia has been found to rapidly elicit robust vasoconstriction S V, 1990, however Zamboni demonstrated that the increased oxygen carriage of hyperoxygenated plasma compensated well for the reduced flow of red blood cells. Additionally, microvascular blood flow in ischemic tissue is in fact improved with HBOT (Zamboni et al., 1993). Further studies showed hyperoxia had beneficial microcirculatory and metabolic effects by limiting the loss of ATP production and decreased accumulation of lactate in ischemic tissue (Cianci et al., 2013). Other researchers have been interested in the consequences of HBOT on CBF, studies using laser doppler flowmetry (Chavko et al., 1998) suggested no systematic changes (Zhang et al., 1995) or small improvements in CBF (Thom et al., 2002) in response to HBOT-induced nNOS activation. However, Sato measured the production of cortical NO during HBOT in rats and found that NO production was improved proportionally to the CBF increase (Sato et al., 2001).

### Cellular Mechanisms

The coenzyme NAD^+^ plays a critical role in cellular bioenergetics and adaptive stress responses. Its depletion has emerged as a fundamental feature of aging (Imai and Guarente, 2014; Kane and Sinclair, 2018) causing defects in nuclear and mitochondrial functions and resulting in many age-associated pathologies (Verdin, 2015) including vascular dementia (Fricker et al., 2018), Alzheimer’s disease (Gong et al., 2013; Hou et al., 2018), and vascular cognitive impairment (Tarantini et al., 2019a; Tarantini et al., 2019b; Kiss et al., 2019). In a recent study mitochondrial NADH redox state were assessed in the brain of awake rats undergoing HBOT treatment. NADH was found to be oxidized to NAD^+^ in parallel to the tissue oxygenation increase, showing maximal tissue oxygenation and greatest accumulation of cellular mitochondrial NAD^+^ after HBOT (Meirovithz et al., 2007). Another study found HBOT significantly increased the level of NAD^+^ 6 h after MCAO, suggesting that the hyperoxygenation exerts a long-lasting effect (Hu et al., 2017). This evidence suggests that treatments aimed at boosting NAD^+^ levels may contribute as a promising avenue to counter age-associated cognitive decline by restoring CBF, improving tissue pO_2_, and ameliorating cellular bioenergetics in the brain (Dave et al., 2003; Mouchiroud et al., 2013; Rajman et al., 2018).

A growing body of evidence implicates bioenergetic deficits, mitochondrial dysfunction, and impaired reduction‐oxidation (redox) homeostasis in the age-related development of neurodegenerative diseases (Mandal et al., 2012; Venkateshappa et al., 2012; Grimm and Eckert, 2017) and cognitive impairment (Mandal et al., 2012). Studies investigating the effects of HBOT on mitochondrial function (Dave et al., 2003) and cellular energetics in endothelial progenitor cells (Hauer-Jensen, 2017; Hsu et al., 2019) and neurons (Yang et al., 2016) have shown that HBOT significantly modulates the basal respiration, improved respiratory capacity, and increased mitochondrial mass following a single HBOT session. Bullock et al. (Zhou et al., 2007) found that hypoxia alone increased cerebral ATP levels in rats, while HBOT improved cognitive recovery and reduced the loss of hippocampal neurons following lateral fluid-percussion injury.

Other prominent features of aging are pro-inflammatory phenotypic alterations of cerebral vessels mediated by the decreased activity of Nrf2 (NF-E2-related factor 2), a key redox sensitive transcription factor, which is a key modulator for the expression of antioxidant and detoxicant enzymes, as well as factors involved in repair of oxidative macromolecular damages and other cell survival pathways. Several recent studies have shown that Nrf2 activity exerts multifaceted anti-aging vasoprotective effects against the pathogenesis of age-related vascular cognitive impairment (Ungvari et al., 2010; Ungvari et al., 2011a; Valcarcel-Ares et al., 2012; Tarantini et al., 2018a). Recent studies administering HBOT in isolated human microvascular endothelial cells (Godman et al., 2010) and diabetic mice (Verma et al., 2015) have identified HBOT as a Nrf2 activator and -mediated oxidative stress response as one of the primary responders to HBOT. HBOT has also been widely used as a treatment adjunct for vascular disease, and in addition to increasing oxygen delivery to the marginally perfused ischemic/hypoxic tissues, HBOT has also been shown to promote angiogenesis and improve cellular metabolism that has been impaired by hypoxia while significantly reducing post-ischemic edema, an effect that persists after treatment (Goyal et al., 2019). Dietary habits leading to metabolic stress and aging have been linked to a decline in microvascular density both in the brain of mouse models of aging (Balasubramanian et al., 2020).

In a recent study the measured hippocampal microvascular rarefaction and the loss of hippocampal-dependent cognitive function positively correlated (Tucsek et al., 2014). Endothelial cells lining the brain microvasculature have been found to be particularly sensitive to these stressors (Ungvari et al., 2011b) and could be implicated in the age-dependent loss of neurovascular coupling responses (Tarantini et al., 2017c; Tarantini et al., 2018b; Tarantini et al., 2019a; Levit et al., 2020), and brain microvascular rarefaction (Tucsek et al., 2014). Additionally, HBOT-induced angiogenesis and fibroplasia has been shown to promote healing to radiated tissue. This is especially useful because radiated tissue does not spontaneously revascularize due to their unique wounding pattern (Goyal et al., 2019). Aged mice exhibit vascular endothelial growth factor (VEGF) signaling insufficiency (Grunewald et al., 2021). At the molecular level, increase in VEGF production mediates the effects of HBOT on angiogenesis. Several mechanisms have been put forth to explain how HBOT induces VEGF signaling to promote angiogenesis. The first mechanism involves increase in VEGF at the transcriptional level which is mediated by HBOT-induced binding of the transcription factor AP-1 to VEGF promoter. *In vitro* studies in human umbilical endothelial cells show that stress-activated protein kinase/c-June N-terminal kinase (SAPK/JNK) pathway and the extracellular signal-regulated kinase (ERK) pathway are involved in HBOT mediated increase in VEGF transcription through AP-1 (Lee et al., 2006). The second mechanism involves HIF-1α mediated VEGF induction. The intermittent normoxic periods between the HBOT session is sensed as hypoxia and results in increase in HIF1α which subsequently induces VEGF expression and angiogenesis (Cimino et al., 1985).

In summary HBOT has been showed to have complex effects on oxygen transport and microvascular hemodynamics. The potential beneficial effects of HBOT, such as reduction in hypoxia, decreased edema, pro-angiogenic effects on the microcirculation, and preservation of tissue energetics and metabolism through NAD^+^ repletion should be further investigated to understand their potential therapeutic effects against microvascular mechanisms of vascular aging and the associated loss of cognitive function.

## Experimental Models for HBOT

Although HBOT is available and used worldwide in human medicine, many scientific discoveries that report its beneficial effects for human diseases were first investigated in animal models, primarily rodents (mice, rats) and rabbits but also dogs, cats, and pigs (Edwards, 2010). Current protocols for murine HBOT administration include use of specifically designed homemade devices (Avraham-Lubin et al., 2011; Lu et al., 2016) and commercially available hyperbaric chambers such as the p-1100 from Barotec Hanyuda (Doguchi et al., 2014). To mimic hyperbaric therapy that is administered to humans, mice breathe pure oxygen at pressures ranging from 2 to 4 ATA for 60–90 min daily for at least a week. A minimum of 15 min of pressurization and depressurization is often allowed for animals to adjust to the changes in pressure. Animal models have demonstrated that HBOT creates the necessary oxygen gradients between the hyperoxygenated blood and injured tissues promoting several beneficial effects in multiple pathologies. As the relationship between cerebral microvascular health and cognitive function has become well-recognized (De Silva and Faraci, 2016), great scientific interest is devoted to understanding the potential therapeutic effects HBOT specifically exerts on the brain and its microvasculature.

Studies conducted in animal models suggest that HBOT has powerful beneficial effects in the microvascular endothelial layer of cells lining the small vessels, a recent study demonstrated microcirculatory pro-angiogenic processes are accelerated by HBOT (Knighton et al., 1981; Hunt, 1988; Goyal et al., 2019), and that angiogenesis is promoted through regulation of vascular endothelial growth factor (Johnson and Wilgus, 2014) which then restores tissue pO_2_ within the injured tissue and reestablishes adequate oxygen delivery for regeneration and repair (Hopf et al., 2005; Sheikh et al., 2005; Rodriguez et al., 2008). *In vitro* hyperoxia has been shown to induce endothelial progenitor cells secretion of exosomes which improved the bioactivity of microvascular endothelial cells in the lungs (Zhang et al., 2019). In a study by Giardina et al., genome-wide gene expression microarray analysis in human microvascular endothelial cells revealed that HBOT upregulated genes involved in protein damage control, and identified Nrf2 upregulation as one of the primary consequences following HBOT (Godman et al., 2010). In this study, HBOT induced Nrf2 upregulation and other gene expression changes associated with enhanced endothelial tube formation on Matrigel plates, especially in cells treated twice daily.

Among other conditions, HBOT has shown possible efficacy to treat vascular dementia in experimental models (Xiao et al., 2012). The growing enthusiasm and appreciation for the potential microvascular therapeutic role of experimental hyperoxia is reflected by recent studies aimed at developing models to quantify hyperoxia-driven microvascular changes (Milstein et al., 2016). In a rat model of cerebral ischemia/reperfusion injury it was found that hyperbaric oxygen exposure restored the permeability of the BBB by increasing expression of caveolin-1 and tight junction protein ZO-1 (Li et al., 2018a).

Stimulus-evoked NVC responses are known to decrease with age and with age-associated pathologies (Tarantini et al., 2017b; Tarantini et al., 2017c; Tarantini et al., 2018b; Tarantini et al., 2019a), Lindauer et al. (Lindauer et al., 2010) investigated the role of HBOT in the modulation of NVC responses using laser doppler flowmetry in anesthetized rats equipped with a cranial window and found no HBOT effect on neuronal activity and neurovascular coupling during functional activation (Lindauer et al., 2010). In contrast, a more recent study by Cardenas et al. found that stimulus-evoked BOLD fMRI signals were improved by HBOT in rats (Cardenas et al., 2015). This discrepancy could be accounted by the more superficial cortical layer mesured by doppler versus the deeper layers imaged with fMRI. Interestingly, it was discovered that administration of HBOT for 60 min daily for 14 consecutive days improved pathophysiological and cognitive outcomes in the 3xTg Alzheimer’s disease mouse model by attenuating neuroinflammation (Shapira et al., 2018a; Shapira et al., 2018b). HBOT has been shown to reduce pain using animal models. Early clinical research indicates HBOT may also be useful in modulating human pain; however, further studies are required to determine whether HBOT is a safe and efficacious treatment modality for chronic pain conditions (Sutherland et al., 2016).

Whole-brain irradiation (WBI) is an established model of accelerated aging (Warrington et al., 2013), gamma-irradiation induces senescence in healthy tissues (Csipo et al., 2020) and leads to progressive cognitive dysfunction and gait alterations (Ungvari et al., 2017a). Currently, new strategies are being sought out to preserve cognitive abilities in patients undergoing radiation therapy against brain metastases (Robin and Rusthoven, 2018). Irradiated organs develop hypovascular-hypocellular-hypoxic tissue that does not revascularize spontaneously. In a rabbit model, HBOT demonstrated a dramatic increase in vascular density over both normobaric oxygen and air-breathing control (Marx et al., 1990), suggesting that HBOT may stimulate anti-aging effects by restoring microvascular endothelial function associated with the preservation of cognitive abilities. The presented animal studies expand the field of high-pressure oxygen therapy and provide evidence leading to prospective hypothesis testing for the vasculoprotective role of HBOT, and its contribution to the modulation of cerebral microvascular mechanisms regulating angiogenesis, BBB permeability (Li et al., 2018a), bioenergetics, and reginal modulation of CBF via neurovascular coupling responses and their relationship with cognitive function.

## Clincal Evidence for HBOT

### Vascular Cognitive Impairment

In humans, age-related vascular cognitive impairment accounts for about 30 percent of all cases of dementia, second only to Alzheimer’s disease which accounts for 60 percent (Kalaria et al., 2008). The symptoms of vascular dementia and VCI are consequences of accumulation of age-related vascular phenotypical alterations pathologically affecting the structure and function of the cerebral microvasculature (Jellinger, 2004; Iadecola, 2013; Ungvari et al., 2018a; Ungvari et al., 2018b). Finding preventative and therapeutic prospects remains one of the greatest challenges in the field of geroscience, as many laboratories and investigators have dedicated their efforts to develop therapeutic strategies against VCI (Smith et al., 2017). Growing evidence in animal models has warranted the necessity for human clinical trials to investigate the role of HBOT against VCI in humans (Table 1).

**TABLE 1:** Summary of relevant clinical findings testing the efficacy and safety of HBOT for the treatment of age-related vascular cognitive impairment and dementia. The highlighted studies present existing evidence indicative of a potential therapeutic role for HBOT-induced hyperoxia against age-related cerebromicrovascular pathologies contributing to cognitive impairment, dementia, and decreased healthspan. Vascular clinical evidence for HBOT.

Treatment	Duration	Age	Outcome	References
100% Oxygen by mask at 2 ATA for 90 min with 5-min air breaks every 20 min daily	60 days	>64 y.o.	Increases telomere length Decreases immunosenescence in isolated blood cells	Hachmo et al. (2020)
100% Oxygen at 2.4 ATA, at 37°C for 60 min	Twice/day	Human cells	Increased Nrf2 pathway activation Increased endothelial tube formations on Matrigel	Godman et al. (2010)
100% Oxygen at 2.5 ATA	30 sessions	∼68 y.o.	Gains in post-treatment performance on psychological tests of cognitive functioning	Jacobs et al. (1969)
100% Oxygen at 2 ATA twice daily for 90 min each day	15 days	∼72 y.o	No enhanced cognitive functioning	Raskin et al. (1978)
100% Oxygen by mask at 2 ATA for 90 min with 5-min air breaks every 20 min daily	60 days	∼69 y.o.	Increased cerebral blood flow. Cognitive enhancements in healthy aging adults. Improved attention, information processing speed and executive function	Hadanny et al. (2020)
100% Oxygen at 2.4 ATA for 120 min daily	28 sessions	45 y.o.	Regression of cerebral edema and radionecrosis	Cihan et al. (2009)
100% Oxygen at 2.4 ATA over 8 weeks	30 sessions	20–51 y.o.	No effect on post-concussive symptoms after mild TBI	Wolf et al. (2012)
100% Oxygen by mask at 2 ATA for 45 min	Single exposure	22–68 y.o	Cognitive, motor single tasks, and multitasking performance scores were significantly enhanced	Vadas et al., (2017)
100% Oxygen at 2 ATA for 80 min	5 days/week	18–20 y.o.	Improved memory correlated with enhanced functional connectivity in the left hippocampus	Yu et al. (2015)
100% Oxygen at 2 ATA	4 weeks	n/a	FMD, plasma NO and CGRP significantly increased	Li et al. (2018b)
100% Oxygen at 2 ATA for 60 min 5 days per week	12 weeks	∼68 y.o.	Improved cognitive function in vascular dementia patients	Xu et al. (2019)

The combination of pure oxygen and higher pressure leads to increases in tissue oxygenation while also targeting oxygen and pressure-sensitive genes. The result is restored and enhanced tissue metabolism. Extant studies in rodents demonstrated that, among other effects, HBOT can improve the blood supply and promote neurogenesis in the piriform cortex (Zhang et al., 2010), and in the hippocampus J Y, 2007, to enhance learning and memory, however, the exact mechanisms remain unclear. In humans, the early works performed by Jacobs et al. in 1969 on a group of 13 elderly male patients with a mean age of 68 indicate that intermittent hyperoxygenation can improve cognitive function in the elderly, with the beneficial effects outlasting the duration of the increased pO_2_^112^. In another study performed a few years later on a cohort of 20 elderly individuals, treated with 100 percent oxygen at 2 ATA for a total duration of 15 sessions, some of the treated participants reported increased visual acuity in addition to improvements across a range of cognitive domains (Edwards and Hart, 1974). In these studies, the beneficial effect of hyperbaric oxygen was presumed to be due to improved blood flow, providing compelling evidence of the relationship between restoration of tissue oxygenation and improvement in function.

The clinical evidence for the role of HBOT against cognitive decline in the elderly is not without controversy, in a report, Raskin et al. focused on the psychological and psychomotor test variables administered prior to and following hyperbaric oxygen treatment and failed to detect any cognitive differences as a function of sex, CO2 loading test, or presumed evidence of cerebrovascular disease (Raskin et al., 1978). In 2012 Xiao et al. published a Cochrane review analyzing the effectiveness and safety of HBOT for vascular dementia (Xiao et al., 2012). However, the evidence they presented was insufficient to support HBOT as an effective treatment for patients with vascular dementia since only one study involving 64 patients was included. This work compared HBOT as an adjuvant to donepezil against donepezil alone. Patients receiving HBOT plus donepezil had significantly better cognitive function than the donepezil only group after 12 weeks of treatment, measured by MMSE scoring or by Hasegawa’s Dementia Rating Scale (HDS). A very recent clinical meta-analysis on the efficacy and safety of hyperbaric oxygen as that HBOT can be recommended as an effective and safe complementary therapy for the treatment of vascular dementia (You et al., 2019). This meta-analysis included twenty-five randomized clinical trials and included almost 2,000 patients that underwent HBOT between 2008 and 2017. The results indicated that HBOT markedly improved the Mini-Mental State Examination (MMSE) scores, activities of daily living by Barthel index, and total efficacy rate, while adverse effects were not statistically different between HBOT and control groups (You et al., 2019). Subgroup analysis revealed that 7–8 weeks of 60 min HBOT administration produce the maximum therapeutic effect to VCI patients, and the positive outcomes are more precise and reliable within this administration regimen. To provide some mechanistic insight, a recent study published this year from Xu et al. included a total of 158 patients with vascular dementia which were randomly divided into control and hyperbaric oxygen groups (Xu et al., 2019). HBOT was administered 5 days per week for 12 weeks, with each session lasting 60 min each at 2 ATA 100% O_2_. The findings from Xu et al. concluded that after the treatment period, patients receiving HBOT not only showed significantly higher MMSE scores but also exhibited higher serum Humanin levels, compared to control, which highly correlated with MMSE scores. Humanin is a mitochondrial-derived peptide with strong neuroprotective effects (Zárate et al., 2019), which has been found to prevent cognitive decline in clinical and experimental studies (Yen et al., 2018).

HBOT cognitive protection and pro-vascular effects beneficially extend to the vascular endothelium. In a study, 98 patients admitted with chest discomfort were divided in a control group to receive conventional treatment and a hyperbaric oxygen administration group, which received HBOT in addition to conventional treatment. Patients who were administered additional HBOT therapy for 4 weeks exhibited improved flow-mediated vasodilation (FMD) response of the brachial artery, increased plasma nitric oxide (NO), increased calcitonin-gene related peptide (CGRP), and decreased levels of endothelin-1 (ET-1) and high sensitivity C-reactive protein (hsCRP) (Li et al., 2018b). The HBOT-induced improvement in cognitive performance is not limited to individuals suffering from age-related VCI. A prospective, double-blind randomized control, crossover trial including 22 healthy volunteers showed that, compared to the performance at normobaric conditions, both cognitive and motor single tasks scores were significantly enhanced by the hyperbaric oxygen environment (Vadas et al., 2017).

Currently, the U.S. Food and Drug Administration (FDA) and the undersea and hyperbaric medical society (UHMS) are the key agencies providing guidelines and indications for marketing and use of HBOT (Weaver, 2014; Fife et al., 2016). At this time HBOT is approved for the medical treatment of 13 conditions: 1) decompression illness, 2) carbon monoxide poisoning, 3) air or gas embolism, 4) crush injury syndrome, 5) clostridial myositis and myonecrosis, 6) adjunctive treatment of selected problem wounds, 7) chronic refractory osteomyelitis, 8) exceptional blood loss anemia, 9) necrotizing soft-tissue infections, 10) late radiation tissue injury, 11) thermal burns, 12) ischemic skin graft and flaps, and 13) intracranial abscess (Fife et al., 2016). Since, among its proven beneficial effects, HBOT has the unique ability to ameliorate tissue hypoxia, reduce pathologic inflammation, mitigate ischemia reperfusion injury, as well as reduce brain edema, the Department of Defense (DoD) has funded trials to evaluate the use of HBOT in chronic traumatic brain injury (TBI), which thus far is not supported by the evidence (Wolf et al., 2012; Cifu et al., 2014a; Cifu et al., 2014b; Walker et al., 2014; Miller et al., 2015). Patients with TBI may present cognitive deficits within the first 24 h after trauma, in the so-called “acute phase,” which in turn may lead to long-term cognitive impairment and decrease in quality of life. The outcome of this research initiative proved that, in two randomized clinical trials comprised of 143 active-duty or veteran military personnel, composite total scores improved from baseline with administration of HBOT (Weaver et al., 2019).

WBI is a mainstream therapy for patients with both identifiable brain metastases and is associated with significant neurotoxicity. However, it also promotes accelerated senescence in healthy tissues and leads to progressive cognitive dysfunction in up to 50% of tumor patients (Ungvari et al., 2017a). The long-term risk for radiation-induced brain inflammation and necrosis inducing secondary cognitive impairments are increasing concerns. Currently there is no effective treatment for preventing long term radiation-induced brain damage. HBOT is currently indicated as an experimental therapy for patients with suspected radiation-induced neurotoxicity and was proven to reduce further development of radiation damage. In a case report, a 45-year-old man who developed brain radionecrosis in the right frontal and left temporoparietal lobes and, after receiving WBI, was referred to HBOT administration. After HBOT, both clinical and cognitive findings improved, suggesting that akin to experimental results (Warrington et al., 2013), treatments that restore cerebromicrovascular function after WBI-related injuries are associated with improved health outcomes (Cihan et al., 2009).

Duration and HBOT exposure are not standardized, however administration of HBOT for 2 h/day, 5 days/week, for 3 months (Hadanny et al., 2020) was able to produce cognitive improvements. The authors of this recent study measured in 63 healthy, active adults, changes in cognitive function via a standardized battery of comprehensive computerized cognitive assessments, and CBF by functional magnetic resonance imaging. The results showed improved attention, cognitive processing speeds and executive function, adding to the growing body of evidence that HBOT has regenerative effects on the brain (Hadanny et al., 2020). Remarkably, this is the first study to demonstrate the beneficial pro-cognitive effects of HBOT on healthy older subjects (Hadanny et al., 2020), providing evidence for the potential effects of HBOT on the healthspan of aged individuals. Similarly, a double-blind placebo-controlled clinical trial provided additional supporting evidence for the beneficial effects of HBOT by testing the effect of HBOT on brain function and cognitive outcomes in mildly cognitively impaired elderly individuals with diabetes (BenAri et al., 2020). Although there is clinical and experimental evidence in favor of HBOT to improve cognitive function in patients with age-related vascular pathologies, oxygen therapy is not without consequences and should be administered with caution. As such, the mechanism of HBOT warrants further investigation.

Overall, the available evidence suggested that application of HBOT as adjuvant therapy has additional benefits on VCI patients, individuals exposed to WBI, and individuals suffering from TBI, and is generally regarded as safe.

### Other Interesting Clinical Effects of HBOT

Individuals that reside in high-altitude environments are exposed to decreased oxygen tension. Currently, the effects of HBOT on high-altitude dwellers has not been examined, however individuals that are consistently exposed to lower environmental pO2 have shown higher hemoglobin concentration (Mairbäurl et al., 1985). The effects of HBOT on these populations is unclear since during HBOT oxygen can be carried by hyperoxygenated plasma in addition to saturated hemoglobin. An additional interesting clinical finding shows that HBOT could aid in perinatal resuscitation of the newborn with perinatal asphyxia (Yilmaz et al., 2020). Conflicting evidence has shown that excessive oxygen may cause retinopathy or bronchopulmonary dysplasia, providing evidence against the use of HBOT in neonates (Liu et al., 2006). However, HBOT has been used to treat newborns with neonatal hypoxic-ischemic encephalopathy in clinical studies in China. The time window of HBOT is still controversial. In clinical studies, HBOT is usually initiated within one to 7 days after birth, administered one to three times per day at 0.15–0.17 MPa for 60–120 min, and continued for one to four courses of treatment (Liu et al., 2006).

## Adverse Effects of HBOT

HBOT is relatively safe, but this type of treatment does carry some risks, mainly due to the increased pressure and hyperoxia (Jacobs et al., 1969). Most pressure-induced barotrauma is preventable by proper equalization techniques or tympanostomy tubes (Vrabec et al., 1998), and otitis media can be prevented with pseudoephedrine (Brown et al., 1992). More severe barotrauma is rare but may include tympanic rupture, tinnitus, and vertigo. Pulmonary barotrauma and pneumothorax are extremely rare. The hyperoxia poses a fire-hazard (Sheffield and Desautels, 1997), with 77 human fatalities reported from 1923 to 1996. However better practices and improved safety regulations have driven that number down. In North America, from 1968 to 2009, there were no reported deaths related to fire in any facilities operating hyperbaric chambers that complied with the national fire protection association codes.

Some conflicting evidence suggests that high-pressure HBOT may not be for everyone, especially for individuals with uncontrolled pre-existing conditions, such as hypertension. For those individuals a milder pressure might be required. Some studies have reported cerebral vasoconstriction (Lambertsen et al., 1953) and decreased total or regional CBF (Visser et al., 1996; Omae et al., 1998) in healthy volunteers and patients breathing 100 percent O_2_ at 3–4 ATA. However, those transitory fluctuations were driven by the immediate increase in blood O_2_ concentration and CBF was quickly restored. In some studies HBOT increased the production of oxygen free radicals, which can oxidize membrane lipids and proteins, and cause DNA damage (Gill and Bell, 2004) and inhibit bacterial metabolic functions (Memar et al., 2018). Central nervous system (CNS) exposure to high (above 2,000 mmHg) pO_2_ may result in oxygen toxicity, firstly recognized by Paul Bert in 1878 (Kellogg, 1978). NO has been implicated as a mediator for CNS oxygen toxicity through formation of peroxynitrite (ONOO−), however O_2_ toxicity-induced seizures are relatively rare (0.01%) at typical clinical treatment pressures (2 ATA–3 ATA) and are difficult to predict on an individual basis. Oxygen toxicity quickly resolves after withdrawal of oxygen and can be easily mitigated by limiting the duration of HBOT sessions and by providing additional air breaks (Heyboer et al., 2017). HBOT also increases the risk of pulmonary edema in patients with compromised left ventricular function. There are limited published data: two studies reported their incidence at 1 in 1,000 (0.1%) and 1 in 4,500 (0.02%) (Abel et al., 2000; Weaver and Churchill, 2001) While the etiology is not fully known, it appears to be related to hyperbaric oxygen, inducing increased systemic vascular resistance and decreased cardiac output (Whalen et al., 1965; Abel et al., 2000) in this high-risk population (Heyboer et al., 2017).

## Future Directions

Hyperbaric oxygen increases brain pO_2_ by saturating blood oxygen and therefore increasing the volume of oxygenated tissue around small vessels, establishing a steeper O_2_ diffusion gradient between blood and tissue (Demchenko et al., 2005). The combination of pure oxygen and higher pressure leads to increases in brain tissue oxygenation while also targeting oxygen and pressure-sensitive genes, altogether promoting resilience in aging.

No consensus exists indicating the exact adequate clinical levels for achieving medically beneficial results. Nonetheless, supplementary oxygen is routinely administered in patients with adequate oxygen saturation levels with the belief that it will improve oxygen delivery in patients with distressed tissues afflicted by ischemic insults. Importantly, there is newly gathered evidence suggesting that daily 1-h long HBOT administrations for 4–8 weeks may provide beneficial effects against vascular dementia while limiting the adverse effects of transient oxygen toxicity (Table 1). HBOT has been recommended and used for a wide range of medical conditions, with a varying evidence base. The concept, and its therapeutic potential that increased tissue oxygenation can be achieved through increased blood PO_2_ has fascinated physicians and researchers for centuries. Evidence for the widespread use of HBOT for decompression sickness and air embolism is robust and well-proven. The UHMS reviews new evidence and published recommendations for the use of HBOT, over the years the list of indicated conditions which warrant the use of HBOT has increased, and at this time there are 14 conditions for which hyperoxia is a recommended therapy (Weaver, 2014).

Although significant progress has been achieved in identifying the appropriate pathologies for which HBOT could serve a therapeutic role, research efforts should persist in this direction, to advance our understanding of the multifaceted effects of HBOT, as there lays the potential to develop innovative strategies to improve biological endpoints affected by aging. Additionally, understanding how HBOT would affect the cerebral microcirculation in aging to ameliorate vascular health-span and cognitive outcome in the elderly population is of high interest for our aging society. The mechanisms involved in HBOT-induced vasculoprotective effects are multifaceted. Cellular and molecular mechanisms of vascular aging such as BBB permeability, increased inflammation, mitochondrial dysfunction, oxidative stress, loss of Nrf2 activity, and NAD^+^ depletion contribute to the pathogenesis of age-related cerebromicrovascular diseases (Figure 1). Growing evidence presented in this review suggests that HBOT targets these very same processes, ameliorating and reversing microvascular pathologies such as endothelial dysfunction (Godman et al., 2010), microvascular rarefaction (Dhamodharan et al., 2019; Yu et al., 2019), improved blood‐brain‐barrier features (Li et al., 2018a), mitochondrial function (Nukhet Aylin Burns et al., 2018; Lippert and Borlongan, 2019), cellular metabolism, inflammation, and oxidative stress (Efrati and Ben-Jacob, 2014), as well as ameliorating decreased NVC responses (Cardenas et al., 2015) which contribute to the development of age-related neurodegeneration and VCI. Further studies are warranted to explore the cerebromicrovascular effects of HBOT in animal models of aging. If evidence is present to suggest that a well-controlled regimen of hyperoxia would be beneficial to the cerebral microcirculation, it could be hypothesized that such a treatment may be promising as a potential therapy to increase resilience in aging and to delay or ameliorate age-related vascular cognitive impairment and dementias associated with vascular pathologies and impaired cerebral tissue oxygenation.
